# Hymenoplasty in India: A Comprehensive Review of Contemporary Trends and Impact on Young Adults

**DOI:** 10.7759/cureus.41852

**Published:** 2023-07-13

**Authors:** Mitanshu Dhaketa, Sourya Acharya, Avinash B Taksande, Roshan Prasad, Pratiksha K Munjewar, Mayur B Wanjari

**Affiliations:** 1 Preventive Medicine, Jawaharlal Nehru Medical College, Datta Meghe Institute of Higher Education and Research, Wardha, IND; 2 Medicine, Jawaharlal Nehru Medical College, Datta Meghe Institute of Higher Education and Research, Wardha, IND; 3 Physiology, Jawaharlal Nehru Medical College, Datta Meghe Institute of Higher Education and Research, Wardha, IND; 4 Internal Medicine, Jawaharlal Nehru Medical College, Datta Meghe Institute of Higher Education and Research, Wardha, IND; 5 Medical Surgical Nursing, Smt. Radhikabai Meghe Memorial College of Nursing, Datta Meghe Institute of Higher Education and Research, Wardha, IND; 6 Research and Development, Jawaharlal Nehru Medical College, Datta Meghe Institute of Higher Education and Research, Wardha, IND

**Keywords:** cultural variations, psychological impact, young adults, contemporary trends, india, hymenoplasty

## Abstract

Hymenoplasty is a reconstructive surgical procedure aimed at repairing the hymen, and it has gained significant attention in the Indian context due to cultural and societal factors associated with virginity. This review article aims to investigate contemporary patterns of hymenoplasty in India and assess their influence on young adults. The review employed a systematic approach to gather and analyze relevant literature from various academic databases. Selection criteria were established to ensure the inclusion of high-quality studies focusing on hymenoplasty in India. The review provides an overview of the historical context of hymenoplasty, highlighting cultural and societal factors that influence the practice. It also examines the evolution of attitudes and perceptions regarding virginity and the hymen in India. Contemporary trends in hymenoplasty are analyzed, including the prevalence and frequency of procedures, demographic factors, patient profiles, popular motivations, and variations in surgical techniques. The socio-cultural impact on young adults is explored, emphasizing the psychological and emotional implications, the influence of societal norms and family expectations, gender dynamics, and the stigma and societal judgment that individuals face. The article concludes with recommendations for future research, including evaluating long-term outcomes and psychological well-being, exploring cultural and regional variations, assessing counseling services, and further examining ethical considerations and professional guidelines. Furthermore, the importance of comprehensive sex education, open dialogue, and discussions regarding virginity and sexual health are highlighted as crucial steps toward creating a more informed and empathetic society.

## Introduction and background

Hymenoplasty is a surgical procedure that reconstructs or repairs the hymen, a thin membrane located at the entrance of the vaginal canal. Traditionally associated with virginity, hymenoplasty has gained significant attention in recent years due to its cultural, social, and psychological implications. While the procedure is not exclusive to India, it holds particular significance in the Indian context, where cultural and societal norms surrounding virginity are deeply ingrained [1].

India is a diverse country with a rich cultural heritage where concepts of purity, honor, and virginity are important. The association of the hymen with virginity and societal expectations can exert tremendous pressure on young individuals. Hymenoplasty has emerged as a response to these expectations, offering a way to reconcile personal choices with societal norms [2,3].

Understanding the contemporary trends and impact of hymenoplasty in India is crucial for several reasons. First, it sheds light on the evolving dynamics of sexuality, gender, and identity in a rapidly changing society. Second, it highlights the influence of cultural and social factors on individual decision-making and mental well-being. Lastly, studying hymenoplasty can contribute to broader conversations about reproductive rights, bodily autonomy, and the need for comprehensive sexual education [4-6].

This review article provides a comprehensive analysis of contemporary trends and the impact of hymenoplasty on young adults in India. This review examines existing literature and research to explore the motivations, practices, and consequences associated with hymenoplasty. Through this review, we hope to contribute to a better understand of what is a complex social and medical phenomenon. By addressing the significance and implications of this practice, we aim to facilitate informed discussions, raise awareness, and provide insights for healthcare providers, policymakers, and society at large.

## Review

Methodology

**Figure 1:**
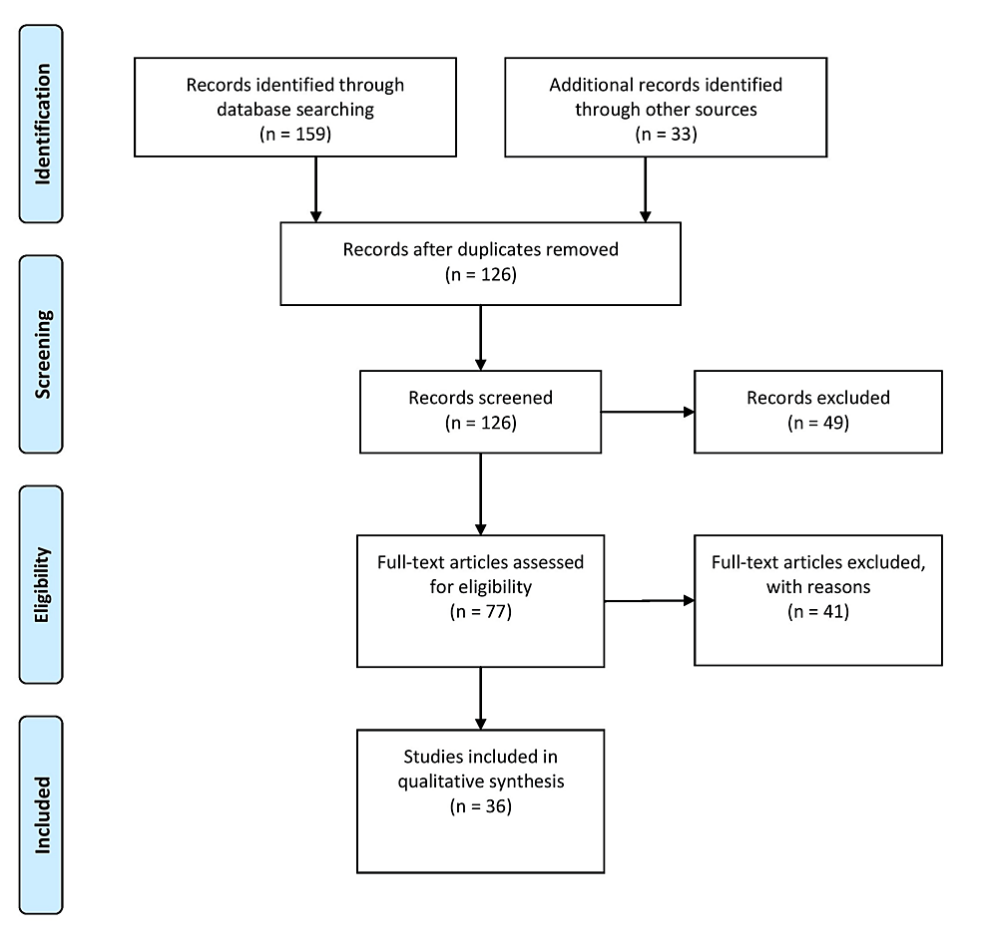
The selection process of articles used in this study. Adopted from the Preferred Reporting Items for Systematic Reviews and Meta-Analyses (PRISMA).

This comprehensive review employed a systematic approach to gather and analyze relevant literature on hymenoplasty in the Indian context. The research encompassed qualitative and quantitative research studies and academic papers to ensure a comprehensive understanding of the subject matter. An extensive search was conducted across multiple academic databases, including PubMed, Scopus, and Google Scholar, to identify relevant sources. The search utilized pertinent keywords and phrases such as "hymenoplasty," "virginity surgery," "reconstructive surgery," and "India." By employing this systematic research approach, a wide range of scholarly materials was accessed and reviewed to provide a comprehensive overview of the topic. Maintaining the rigor and relevance of the review necessitated the establishment of specific selection criteria for the inclusion of studies. These criteria encompassed several key aspects. Firstly, studies conducted in India or focusing specifically on hymenoplasty in the Indian context were prioritized. Additionally, preference was given to studies published in peer-reviewed journals, conference proceedings, and reputable sources. Studies published within a specified timeframe were considered to ensure the incorporation of recent research. Furthermore, studies that provided insights into contemporary trends, motivations, impact, and ethical considerations of hymenoplasty were included. Lastly, studies in English or with comprehensive English summaries were preferred to facilitate analysis and interpretation. By adhering to these selection criteria, the review ensured the inclusion of high-quality research that directly addressed the study's objectives. Figure 1 describes the selection process of articles used in our study.

Historical context of hymenoplasty in India

Overview of Cultural and Societal Factors Influencing Hymenoplasty

Hymenoplasty does not exist in a vacuum but is deeply intertwined with cultural and societal factors in India. Understanding these influences is crucial to comprehend the motivations behind hymenoplasty. In Indian culture, virginity is traditionally associated with purity, honor, and societal expectations, particularly for women. The intactness of the hymen has been considered a symbol of a woman's chastity and virtue, often linked to concepts of family honor and marital suitability. These cultural norms, combined with the influence of religious beliefs and social pressures, contribute to the demand for hymenoplasty to conform to societal expectations and preserve one's perceived purity [2,7,8].

Evolution of Attitudes and Perceptions Regarding Virginity and Hymen in India

Over time, attitudes and perceptions regarding virginity and the hymen have evolved significantly in India. With the modernization of society and increased exposure to global influences, traditional notions of virginity have faced challenges and scrutiny. Discussions around sexual liberation, individual autonomy, and gender equality have gradually emerged, evaluating the significance placed on the hymen as the sole determinant of a woman's virtue. The younger generation, in particular, is more likely to question and challenge these societal norms, seeking to redefine notions of virginity and navigate a more nuanced understanding of sexuality [9,10].

However, despite these evolving perspectives, the cultural and societal expectations around virginity remain deeply rooted in many communities across India [11]. The pressure to conform to these expectations persists, leading some individuals to seek hymenoplasty to reconcile personal choices with societal demands. The historical context of hymenoplasty in India reflects the enduring influence of cultural beliefs surrounding virginity and the ongoing shifts in attitudes and perceptions driven by societal changes [11,12]. By examining the historical context of hymenoplasty in India, we gain insights into the complex interplay between cultural norms, societal pressures, and the choices made by individuals seeking hymenoplasty. This understanding forms a foundation for further exploration of contemporary trends and the impact of hymenoplasty on young adults in India.

Contemporary trends in hymenoplasty in India

Current Prevalence and Frequency of Hymenoplasty Procedures

Examining the current prevalence and frequency of hymenoplasty procedures in India provides valuable insights into the contemporary trends surrounding this practice. Recent studies and reports suggest that the demand for hymenoplasty has steadily increased in India. Individuals from diverse backgrounds, including urban and rural areas, across different country regions, seek the procedure. Understanding the scale and extent of hymenoplasty in contemporary India helps contextualize its societal impact and shed light on the prevalence of societal expectations surrounding virginity [13,14].

Analysis of Demographic Factors and Patient Profiles

Analyzing demographic factors and patient profiles associated with hymenoplasty reveals important patterns and trends. This analysis may include age, education, socioeconomic status, and geographic location. It helps identify groups or communities more likely to seek hymenoplasty, providing a deeper understanding of the factors influencing this decision. Exploring demographic factors and patient profiles assists in identifying the social, cultural, and economic contexts in which hymenoplasty is more prevalent and offers insights into the intersectionality of individuals seeking this procedure [1].

Popular Motivations and Reasons for Undergoing Hymenoplasty

Understanding the motivations and reasons behind individuals' decisions to undergo hymenoplasty is essential for comprehending societal and personal factors. Various motivations can drive individuals to seek hymenoplasty, including societal pressures, cultural expectations, desires for marriageability, and the need to preserve family honor. Exploring these motivations helps reveal the complex interplay between personal agency, cultural influences, and societal norms. Additionally, examining the motivations and reasons can contribute to discussions on gender roles, sexual autonomy, and the impact of societal expectations on individuals' choices [15].

Variations in Surgical Techniques and Procedures

Hymenoplasty procedures can vary in techniques and approaches. Analyzing the variations in surgical techniques provides insights into the medical aspects of hymenoplasty and the advancements in reconstructive surgery [16]. Different surgical methods, including hymenal repair and regeneration, may be utilized, each with its considerations, benefits, and potential risks. Examining the variations in surgical techniques and procedures helps assess the efficacy, safety, and potential complications associated with hymenoplasty, enabling healthcare professionals to provide informed care and guidance [16].

Socio-cultural impact on young adults

Psychological and Emotional Implications for Young Adults

The socio-cultural impact of hymenoplasty on young adults encompasses significant psychological and emotional implications. The pressure to conform to societal expectations of virginity can lead to anxiety, guilt, and shame for individuals considering or undergoing hymenoplasty. Young adults may experience various emotions, including fear of judgment, internal conflict, and a sense of dissonance between personal desires and societal norms. Understanding the psychological and emotional implications is crucial for providing appropriate support and counseling to young adults navigating these complex dynamics [17,18].

Influence of Societal Norms, Traditions, and Family Expectations

Societal norms, traditions, and family expectations are pivotal in shaping the decision-making process for young adults considering hymenoplasty. In many communities in India, the perception of virginity as a marker of honor and respectability can exert immense pressure on individuals. Young adults often find themselves torn between personal desires and the desire to meet societal expectations. The weight of cultural and family traditions can significantly influence the decision to undergo hymenoplasty, highlighting the need to critically examine the impact of these influences on individual autonomy and agency [19,20].

Gender Dynamics and the Role of Patriarchy in Decision-Making

Gender dynamics and the role of patriarchy are crucial aspects of the socio-cultural impact of hymenoplasty on young adults. Hymenoplasty is deeply intertwined with notions of female sexuality, control, and ownership of women's bodies. The decision to undergo hymenoplasty is often shaped by patriarchal structures that place a premium on women's virginity, reinforcing power imbalances and limiting women's autonomy. Examining gender dynamics and the influence of patriarchy is essential in understanding the societal forces that shape young adults' choices and their implications for gender equality and women's empowerment [21].

Stigma, Shame, and Societal Judgment Faced by Young Adults

Young adults considering or undergoing hymenoplasty often face stigma, shame, and societal judgment. Society's scrutiny and the fear of being labeled as "impure" or "immoral" can be daunting for individuals. The stigma surrounding hymenoplasty can have far-reaching consequences, including social ostracism, strained relationships, and psychological distress. Understanding the stigma and societal judgment faced by young adults is critical for fostering empathy, promoting inclusivity, and challenging the societal norms perpetuating the judgment and discrimination against individuals seeking hymenoplasty [22].

Ethical considerations and legal framework

Examination of Ethical Concerns Surrounding Hymenoplasty

The practice of hymenoplasty raises several ethical concerns that warrant careful consideration. One primary ethical concern revolves around the notion of informed consent. It is crucial to ensure that individuals seeking hymenoplasty fully understand the procedure's risks, benefits, and potential outcomes. The ethical implications of surgical intervention based on societal expectations and pressures must be critically evaluated [23,24].

Another ethical concern is the role of healthcare professionals in providing hymenoplasty services. Medical practitioners must navigate the tension between respecting patients' autonomy and upholding ethical principles such as beneficence and non-maleficence. The ethical obligations of healthcare professionals extend beyond the physical aspects of the procedure and encompass the psychological well-being and long-term consequences for patients [18,25]. Additionally, the commodification of virginity raises ethical questions. The emphasis on reconstructing the hymen to satisfy societal expectations perpetuates the objectification and commodification of women's bodies. Examining these ethical concerns helps foster a broader understanding of the complex dynamics surrounding hymenoplasty.

Legal Regulations and Policies Governing Hymenoplasty in India

In India, legal regulations and policies regarding hymenoplasty are important to consider. Currently, there are no specific laws addressing hymenoplasty directly. However, the procedure may fall under the broader framework of medical ethics, patient rights, and professional conduct regulations [7,23,26].

Medical professionals are guided by ethical guidelines and codes of conduct established by medical associations and regulatory bodies. These guidelines emphasize the importance of informed consent, patient confidentiality, and healthcare professionals' responsibility to prioritize their patient's best interests [27]. It is essential to examine the existing legal framework and its adequacy in addressing the ethical concerns surrounding hymenoplasty. Assessing the gaps and potential areas for improvement in the legal and regulatory landscape helps shape policies that safeguard patients' rights, promote ethical practices, and ensure comprehensive care.

Psychological support and counseling

Importance of Pre- and Post-Operative Psychological Support

Psychological support is crucial for individuals considering and undergoing hymenoplasty before and after the procedure. Pre-operative psychological support helps individuals make informed decisions by providing a safe space to explore their motivations, expectations, and concerns. It allows for an open and non-judgmental dialogue, enabling individuals to reflect on the societal pressures, cultural influences, and personal factors influencing their decision [28].

Post-operative psychological support is equally important as individuals navigate the emotional and psychological aftermath of the procedure. It helps individuals process their experiences, manage any feelings of guilt or shame, and address potential challenges that may arise, such as societal judgment or strained relationships. Post-operative psychological support can contribute to empowerment, self-acceptance, and improved emotional well-being [29].

Role of Counseling Services in Managing Expectations and Psychological Well-Being

Counseling services are crucial in managing expectations and promoting psychological well-being for individuals considering or undergoing hymenoplasty. Counseling provides a supportive and non-judgmental environment where individuals can express their concerns, explore their motivations, and gain a deeper understanding of the implications of their decision [30].

Counselors can help individuals develop realistic expectations about the outcomes of hymenoplasty, ensuring that they understand the limitations and potential risks involved. They can also address misconceptions and myths surrounding virginity, challenging societal norms and promoting a more nuanced understanding of sexuality [31].

Furthermore, counseling services can assist individuals in building resilience, coping mechanisms, and self-esteem, helping them navigate the potential stigma and societal judgment they may face. Counseling sessions may involve techniques such as cognitive-behavioral therapy, supportive counseling, and psychoeducation to address the decision-making process's psychological and emotional aspects and the procedure's aftermath [32]. The role of counseling services goes beyond providing support to individuals. They also raise awareness about the societal pressures and cultural influences surrounding hymenoplasty. By engaging in community education and awareness programs, counseling services can foster discussions on gender equality, sexual autonomy, and respecting individual choices [33].

Future directions and recommendations

Areas for Further Research and Investigation

While significant progress has been made in understanding hymenoplasty in the Indian context, several areas warrant further research and investigation. Some potential areas for future research include the following.

Long-term outcomes and psychological well-being: It is important to conduct research that focuses on the long-term psychological impact of hymenoplasty on individuals. This research should explore factors such as self-esteem, body image, and sexual satisfaction to understand better how hymenoplasty affects individuals' overall well-being beyond the immediate post-operative period. Longitudinal studies that follow individuals over an extended period can provide valuable insights into the psychological implications and potentially long-lasting effects of hymenoplasty [4,29].

Cultural and regional variations: Investigating the cultural and regional variations in attitudes, motivations, and prevalence of hymenoplasty can enhance our understanding of this practice within different communities and geographic locations in India. By exploring how cultural norms, traditions, and societal expectations influence the demand for hymenoplasty, we can gain insights into the diverse factors contributing to the decision-making process. This research can also shed light on the impact of regional socio-cultural factors on the prevalence and acceptance of hymenoplasty, allowing for targeted interventions and education efforts [6].

Evaluation of counseling and support services: Research should assess the effectiveness of counseling and support services in assisting individuals before and after hymenoplasty. This evaluation should go beyond satisfaction surveys and measure outcomes such as emotional well-being, empowerment, and the ability to make informed decisions. Understanding the impact of counseling and support services can guide the development of evidence-based interventions that address the unique needs of individuals considering or undergoing hymenoplasty. This research can also inform the training and guidelines for healthcare professionals, ensuring they are equipped to provide comprehensive care that addresses the psychological and emotional aspects of hymenoplasty [34].

Ethical considerations and professional guidelines: Further exploration of the ethical concerns surrounding hymenoplasty and developing professional guidelines are essential to navigate this procedure's complex ethical landscape. Research should delve into the ethical implications of performing hymenoplasty, including issues of informed consent, patient autonomy, and the objectification of women's bodies. Examining the existing ethical guidelines and regulations and engaging in ethical discussions can enhance the ethical framework for healthcare professionals involved in hymenoplasty. This research can also contribute to developing comprehensive professional guidelines that promote ethical practices and patient-centered care [5].

Advocacy for Comprehensive Sex Education and Awareness

Promoting comprehensive sex education and awareness is crucial in addressing the underlying issues surrounding hymenoplasty. By incorporating comprehensive sex education in schools and educational institutions, young adults can gain accurate information about virginity, sexuality, and reproductive health. This education should promote healthy attitudes toward sexuality, challenge harmful societal norms, and foster respect for individual choices [35,36]. Advocacy efforts can also target policymakers and stakeholders to emphasize the importance of comprehensive sex education to empower young adults, promote sexual health, and address the pressures contributing to the demand for hymenoplasty [35,36].

Importance of Open Dialogue and Discussions Regarding Virginity and Sexual Health

Encouraging open dialogue and discussions surrounding virginity and sexual health is crucial for dismantling societal taboos and promoting a more inclusive and understanding society. Creating safe spaces for conversations, both online and offline, can facilitate a deeper understanding of the complexities surrounding hymenoplasty, foster empathy, and challenge societal judgments [5,14,35]. By engaging in open dialogue, individuals can share their experiences, challenge stereotypes, and promote a broader understanding of sexual autonomy, consent, and personal choices. This dialogue should involve diverse stakeholders, including healthcare professionals, educators, policymakers, and community leaders, to ensure a comprehensive and inclusive approach [6].

## Conclusions

The findings of this review have important implications for healthcare providers, policymakers, and society. Healthcare providers should recognize the psychological and emotional implications of hymenoplasty and provide comprehensive care that includes pre- and post-operative psychological support. They should also adhere to ethical guidelines and foster open communication with patients to ensure informed consent and patient-centered care. Policymakers can use the findings to develop regulations and policies that protect the rights of individuals seeking hymenoplasty while addressing ethical concerns and ensuring quality healthcare services. Advocacy for comprehensive sex education and awareness can help challenge societal norms and promote a more inclusive and informed approach to sexual health. Society should engage in open dialogue and discussions surrounding virginity, sexual health, and the complexities of hymenoplasty. By promoting empathy, understanding, and respect for individual choices, we can create a more supportive and accepting environment for individuals navigating the decision-making process and the aftermath of hymenoplasty.
